# Widely Targeted Metabolomics Analysis of the Changes to Key Non-volatile Taste Components in *Stropharia rugosoannulata* Under Different Drying Methods

**DOI:** 10.3389/fnut.2022.884400

**Published:** 2022-05-19

**Authors:** Yi Liu, Fangbo Meng, Pengyu Tang, Daomei Huang, Qixing Li, Mao Lin

**Affiliations:** ^1^Institute of Agricultural Products Processing, Guizhou Academy of Agricultural Sciences, Guiyang, China; ^2^Guizhou Vocational College of Foodstuff Engineering, Guiyang, China; ^3^Guizhou Characteristic Food Technology Co., Ltd, Guiyang, China

**Keywords:** drying methods, formation mechanism, non-volatile taste components, stropharia rugosoannulata, widely targeted metabolomics

## Abstract

*Stropharia rugosoannulata* is an extremely perishable edible fungi product, and drying can delay its deterioration, however, drying will affect its flavor, especially the non-volatile taste substances dominated by amino acids, nucleotides, organic acids and carbohydrates. Currently, which drying method is the most suitable for the drying of *S. rugosoannulata* remains unknown, we need to fully consider the economic efficiency of the method and the impact on flavor. But we have limited comprehensive knowledge of the changed non-volatile taste metabolites as caused by drying processes. Here, an LC-MS/MS-based widely targeted metabolome analysis was conducted to investigate the transformation mechanism of *S. rugosoannulata* non-volatile taste components after undergoing hot air drying (HAD), vacuum freeze drying (VFD), and microwave vacuum drying (MVD). A total of 826 metabolites were identified, 89 of which—48 amino acids, 25 nucleotides, 8 organic acids, and 8 carbohydrates—were related to non-volatile taste. The drying method used and the parts of *S. rugosoannulata* (stipe and pileus) influenced the differences found in these metabolites. The possible mechanisms responsible for such chemical alterations by different drying methods were also investigated by a Kyoto Encyclopedia of Genes and Genomes (KEGG) pathway analysis. Amino acid metabolism (alanine, aspartate, and glutamate metabolism; glycine, serine, and threonine metabolism; arginine and proline metabolism; valine, leucine, and isoleucine biosynthesis) was the main metabolic pathway involved. Pathway enrichment analysis also identified differences in non-volatile taste components among three drying methods that may be closely related to the applied drying temperature. Altogether, the results indicated that as an economical and convenient drying method, HAD is conducive to improving the flavor of *S. rugosoannulata* and thus it harbors promising potential for practical applications.

## Introduction

*Stropharia rugosoannulata* is a precious edible mushroom that has been widely cultivated in many countries, but especially in Southwest China (1). Several studies reported that *S. rugosoannulata* harbors various proteins, polysaccharides, phenols, mineral elements (2) in addition to several bioactive compounds whose benefits include antioxidant (3), antitumor (4), anti-diabetic (5), anti-viral (6) properties, and so forth. In addition, there are some non-volatile flavor components, such as free amino acids, organic acids, nucleotides, which can produce an umami taste that is among the most prominent characteristics of mushrooms (7). In recent years, because of its texture, rich nutrition, and unique flavor, it has quickly become one of the world’s best-selling mushrooms (2).

However, fresh *S. rugosoannulata* are prone to rapid spoilage and deterioration due to microbial and physical factors, causing incalculable damage to its economic value (8). Among the various techniques used to extend its shelf-life, dehydration is the most effective preservation method for this prized mushroom (9). The three main dehydration methods are hot air drying (HAD), vacuum freeze drying (VFD), and microwave vacuum drying (MVD), which are now the most popular dehydration techniques applied to mushrooms (10). However, drying can effect the taste components, appearance, and nutrients of the mushrooms (11). We should therefore fully consider both practical and economic aspects when choosing a suitable drying method for *S. rugosoannulata.*

Because of its easy operation and low cost, HAD is the most frequently applied mode of preservation worldwide (12). As pointed out by many researchers, exposure to higher temperatures can induce the Maillard reaction and thereby affect flavor compounds (13). For example, HAD technology can enhance the umami taste of mushrooms by increasing the content of organic acids (14) and amino acids (15, 16). However, a heat treatment that lasts too long can seriously damage other characteristic flavor substances and concentration of key nutrients of the dried mushroom (14, 15).

Compared with HAD, VFD can minimize degradation of the mushrooms’ distinctive aroma (17, 18). However, due to its energy consumption, the promotion of the VFD technique is generally a problem (19). VFD can maintain the good appearance and nutritional value of mushrooms (20), and MVD has the greater capacity to produce more amino acids (9). However, the differential changes in nutrients and taste chemicals of mushrooms caused by HAD, VFD, and WVD remain largely unexplored, and there are few reports on the effects of drying on non-volatile taste compounds and metabolites in *S. rugosoannulata.*

In recent years, widely targeted metabolomics has been applied to the field of non-volatile compounds and nutrients in food (21). This technique combines the advantage of non-target and target metabolite detection technologies, thereby achieving high throughput while exhibiting high sensitivity and wide coverage of detection of metabolites in foods and plants (22). Zou et al. (23)used this technique to research the key taste components in two wild edible *Boletus* mushrooms. Therefore, we considered that widely targeted metabolomics could be applied to the comprehensive detection and analysis of nutrients and non-volatile taste components in *S. rugosoannulata* mushrooms.

The aim of this study was to better understand the changes in each category of main non-volatile taste metabolites of *S. rugosoannulata* in response to different drying methods. To do this, we used ultra performance liquid chromatography–tandem mass spectrometry (UPLC–MS/MS) combined with a widely targeted metabolomics technology to detect the non-volatile metabolites, including amino acids, nucleotides, organic acids, and sugars. Further, possible metabolic pathways were predicted through the transformation of metabolites. We anticipate our results will provide a theoretical reference and objective basis for the formation mechanism of the umami taste in dried mushrooms of *S. rugosoannulata* and perhaps similar ones.

## Materials and Methods

### Raw Material

*Stropharia rugosoannulata* was collected in April 2021 from the Horticultural Institute of Guizhou (106°39″59N, 26°30″20E). All samples were immediately transferred to the lab for their pretreatment: surface mud and stipe root were wiped off.

### Drying Methods

#### Microwave Vacuum Drying

Roughly 2,000 *g* of samples were dried inside the Sort Cleverly Inactive Microwave Vacuum Dryer (XINQI, Guiyang, Guizhou, China) with three progressive methods, as follows: Method 1, MW control was 2000 W for 30 min; Method 2, Mw control was 1000 W for 10 min; Method3, Mw control was 500 W for 10 min.

#### Hot Air Drying

The samples were spread on plates and these placed in a drying chambers (HT-KRZH-1IV Drier, Languanda, Guiyang, Guizhou, China)with modified humidifying and warming treatments applied as follows: Strategy 1, temperature of 55°C and 50% humidity for 6 h; Strategy 2, temperature of 45°C and 40% humidity for 8 h; Strategy 3, temperature of 35°C and 30%humidity for 12 h.

#### Vacuum Freeze Drying

The samples were quick-frozen at –40°C within the TDE400 ultra cold capacity freezer (ThermoFisher Logical, Beijing, China). Their solidification was halted after 12 h, then the pre-frozen tests were spread on plates and dried by a FD-1D-50 Vaccum Solidify Drier (BIOCOOL, Beijing, China).

### Metabolites Analysis

#### Metabolites’ Extraction

The freeze-dried samples were fragmented in a blender run for 30 s at 60 Hz. Then a 50-mg aliquot of individual samples were exactly weighed and put into an Eppendorf tube, after expansion of 700 μL of extricate arrangement (methanol/water = 3:1, precooled at –40°C, containing an internal standard). After 30 s of vortexing, the tests were homogenized at 40 Hz for 4 min and sonicated for 5 min in an ice-water shower. They were homogenized and sonicated again (three times), after which the samples were extricated overnight at 4°C on a shaker. Next, they were centrifuged at 13,800 × *g* for 15 min at 4°C. The supernatant was carefully filtered through a 0.22 μm microporous membrane, then the resulting supernatants were diluted 20 times with extract solution containing internal standard and vortexed for 30 s and transferred to 2 mL glass vials, and take 30 μL from each sample and pooling as QC samples. Store at –80°C until the ultra performance liquid chromatography-tandem mass spectrometry (UHPLC-MS) analysis.

#### Ultra Performance Liquid Chromatography-Tandem Mass Spectrometry Analysis

The UHPLC separation was carried out using an EXIONLC System (Sciex Technologies, Framinghan, MA, United States). The mobile phase A was 0.1% formic acid in water, and the mobile phase B was acetonitrile. The column temperature was set to 40°C. The auto-sampler temperature was set at 4°C and the injection volume was 2 μL.

A Sciex QTrap 6500 + LC-MS/MS system (Sciex Technologies, Framinghan, MA, United States) was used for the assay development. Typical ion source parameters were as follows: ionspray voltage: +5,500/–4,500 V, curtain gas: 35 psi, temperature: 400°C, ion source gas 1:60 psi, ion source gas 2: 60 psi, DP: ± 100 V.

#### MS Data and Statistical Analysis

SCIEX Analyst Work Station software (v1.6.3) (Sciex Technologies, Framinghan, MA, United States). was employed for the MRM data acquisition and processing. The MS raw data (.wiff) files were converted into the TXT format, using MSconventer. R program and database were applied to obtain peak detections and to carry out their annotation. The quantitative analysis of metabolites was performed using MRM analysis of QQQ mass spectrometry. After obtaining metabolite mass spectrometry data of different samples, peak area integration was performed on the mass spectrum peaks of all the substances, and the mass spectrum peaks of the same metabolite in different samples were integrated for correction.

The SIMCA (V16.0.2, Satorious Stedim Data Analytics AB, Umea, Sweden) software is used to perform Par format processing on the data, and then conduct automatic modeling and analysis to form PCA model In this project. We screened differential metabolites of *S. rugosoannulata* using the p-value of student’s test is less than 0.05, and the threshold variable importance in projection (VIP) value (VIP ≥ 1) from the OPLS-DA model. The screening of different metabolites was visualized in the figure of the volcano plot. KEGG were used for enrichment analysis of differential metabolites and finding metabolic pathways.

## Results and Discussion

### Metabolites Under Different Drying Methods of *Stropharia rugosoannulata*

**FIGURE 1:**
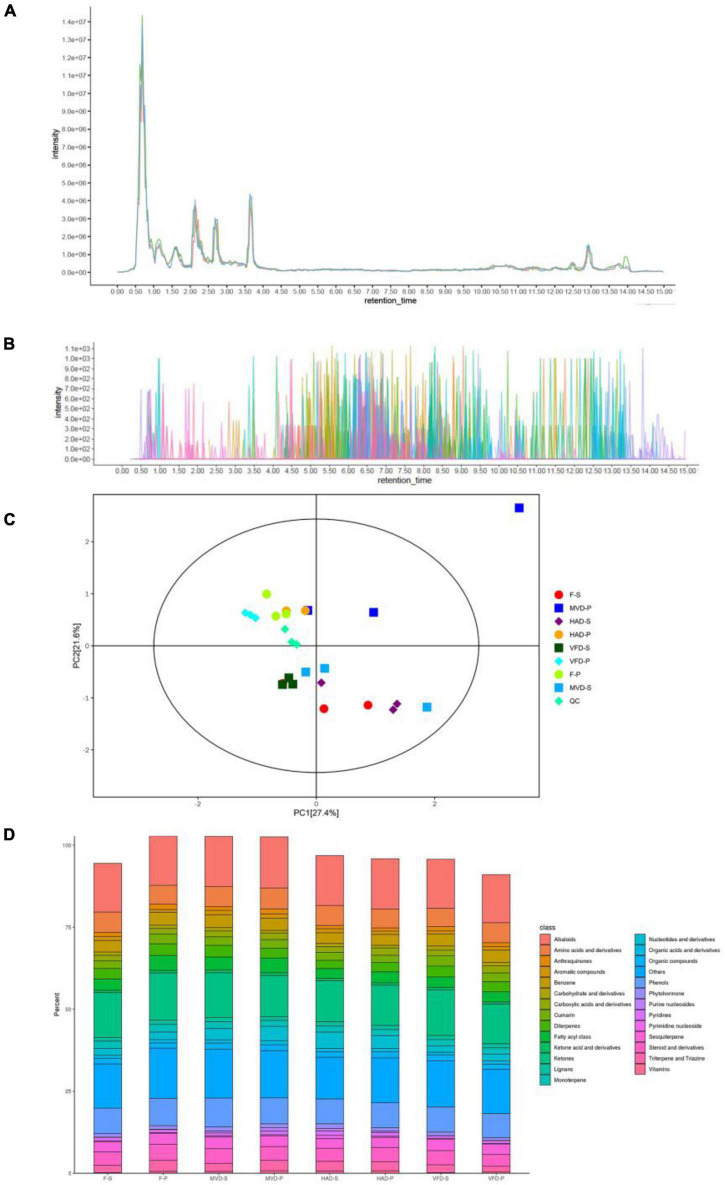
**(A)** Total ion current of one quality control sample as revealed by mass spectrometry detection, and **(B)** a multi-peak detection plot of the sample metabolites in the multiple reaction monitoring mode. **(C)** PCA analysis of metabolites identified from the stipe and pileus of *S. rugosoannulata* mushrooms subjected to different drying methods. **(D)** Relative content of principal components. F-S, fresh stipe; F-P, fresh pileus; MVD-S, microwave vacuum drying of stipe; MVD-P, microwave vacuum drying of pileus; HAD-S, hot air drying of stipe; HAD-P, hot air drying of pileus; VFD-S, vacuum freeze drying of stipe; VFD-P, vacuum freeze drying of pileus.

To investigate the chemical composition profile in the stipe and pileus of *S. rugosoannulata* under different drying methods, the metabolites were identified by LC-MS/MS analysis. The overlay analysis of the QC-TIC diagram (Figure 1A) and the sample multi-peak detection of QC-EIC diagram (Figure 1B) showed that the obtained data had robust reliability and repeatability. As Figure 1C shows, we carried out a principal components analysis (PCA) on metabolites. Whether left fresh or treated with different drying methods, the pileus samples are positioned on the upper side of the figure, while those of stipe are on the bottom. This result indicates that pileus and stipe metabolites are quite different chemically. Similar observations were reported previously (2). Furthermore, samples of different treatments are located separately in the figure, indicating that the drying methods can affect the metabolites in *S. rugosoannulata.*

As seen in Figure 1D, 826 metabolites were divided into 25 classes, including 123 alkaloids,105 ketones, 63 phenols, 48 amino acids and derivatives, 30 benzenes, 29 fatty acyl class, 26 sesquiterpenes, 25 nucleotides and derivatives, 25 diterpenes, 20 triterpenes and triazines, 18 cumarins, 17 monoterpenes, 13 organic compounds, 10 anthraquinones, 9 aromatic compounds, 9 phytohormones, 9 lignans, 8 carbohydrate and derivatives, 8 organic acids and derivatives, 8 pyridines, 6 ketone acid and derivatives, 4 vitamins, 3 pyrimidine nucleosides, 3 purine nucleosides, and others. The main metabolites of *S. rugosoannulata* were differentially changed by the drying mode, including alkaloids, phenols, as well as some non-volatile flavor substances.

### Key Non-volatile Taste Components Under Different Drying Methods of *Stropharia rugosoannulata*

**FIGURE 2:**
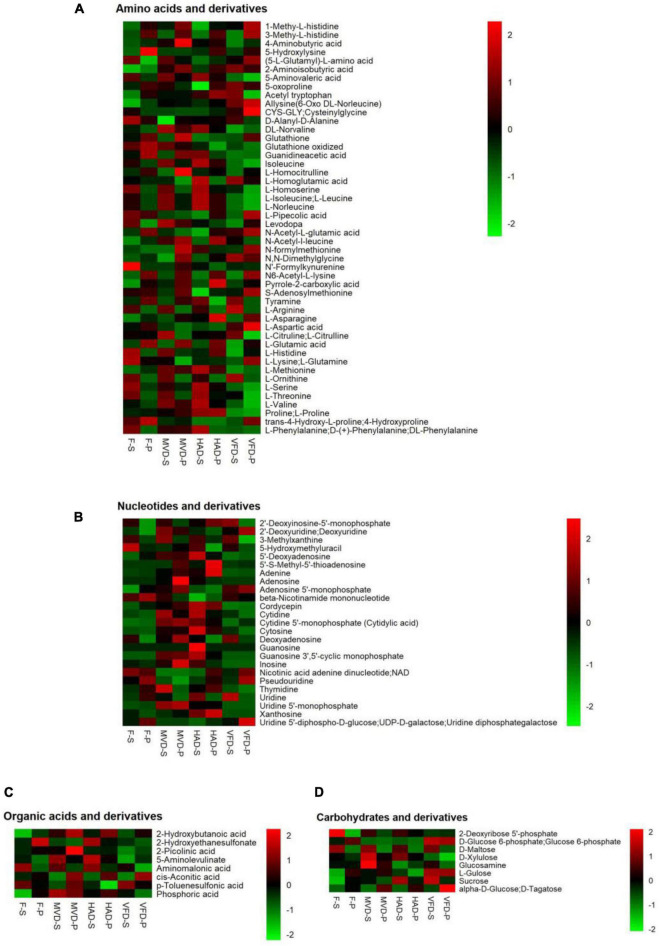
Heat maps of the levels of the non-volatile metabolites under different drying methods. The abscissa represents different experimental groups, the ordinate represents the different metabolites compared by the group. The color indicates the relative expression of metabolites, from low (green) to high (red). **(A)** Amino acids and their derivatives, **(B)** nucleotides and their derivatives, **(C)** organic acid and their derivatives, and **(D)** carbohydrates and derivatives.

To clarify the influence of drying methods on *S. rugosoannulata*’s non-volatile taste compounds, we focused on classes of metabolites likely to be major contributors to umami taste. In Figure 2 are heat maps presenting the main components that affect umami taste, including amino acids and their derivatives, nucleotides and their derivatives, organic acids and their derivatives, carbohydrate and derivatives. These results are described below.

#### Amino Acids and Their Derivatives

This study detected 48 amino acids and their derivatives. As evinced by Figure 2A, their total amounts in stipe followed this trend in rank: HAD-S > F-S > MVD-S > VFD-S, which differed from that for pileus: HAD-P > MVD-P > VFD-P > F-P. Amino acids are one of the most important taste active compounds in mushrooms. Based on their taste characteristics, amino acids are chiefly divided into umami, sweet, bitter, and tasteless categories (24). We found greater quantities of 27 amino acids and their derivatives in stipe after HAD than either MVD or VFD, and likewise for 24 of them in pileus. In particular, the L-aspartic acid, L-glutamic acid, L-lysine, L-glutamine, L-threonine, L-serine, L-histidine, L-valine, and L-methionine content of the stipe and pileus after undergoing HAD are higher vis-à-vis MVD and VFD. Similar results were found in two previous studies (10, 14). Asp and Glu are typical MSG-like components in edible fungi, Thr and Ser are sweet components, and Val and Leu are bitter components (25). Our results show that hot air drying can improve both umami and sweet amino acids, but at the same time it would also produce more bitter amino acids. Among the latter, 4-guanidinobutyric acid (GABA) has been reported in other research (23).

#### Nucleotides and Their Derivatives

Besides umami acids, the nucleotides in edible fungi also greatly influence their taste (26). Through research on fresh and dried stipe and pileus samples, we uncovered 25 nucleotides and their derivatives (Figure 2B). Among the three drying methods, after HAD the total content of nucleotides and their derivatives were highest by, even higher than those of fresh stipe and pileus samples of *S. rugosoannulata*. This result is not unlike that of a previously published study (18) and may be explained by thermal decomposition and enzymatic hydrolysis of ribonucleic acids or deoxyribonucleic acids during the hot air drying process (25).

#### Organic Acids and Their Derivatives

Organic acids impart sourness and astringency independently, hence they also contribute to edible fungi’s complex and unique taste (27, 28). By determining the metabolites in *S. rugosoannulata*, eight kinds of organic acids and their derivatives were found. Unlike for its amino acids and nucleotides, the total amount of organic acids in the stipe and pileus was greatest after their MVD in comparison with the other treatments, showing this trend in rank: MVD > HAD > F > VFD.

#### Carbohydrates and Their Derivatives

Research has shown that sugars can produce sweetness and are the main ingredient that determines the taste of edible fungi (29). Here, eight carbohydrates and their derivatives, chiefly alpha-D-glucose, glucose 6-phosphate, D-maltose, L-gulose sucrose, were found (Figure 2D). The total content carbohydrates in VFD exceeded those in MVD, perhaps due to the Maillard reaction which may affect the carbohydrate content (30). But given that we found a higher carbohydrate content in HAD, this could instead be attributable to the increased dry matter content after HAD.

### Screening and Analysis of Differential Metabolites

**FIGURE 3:**
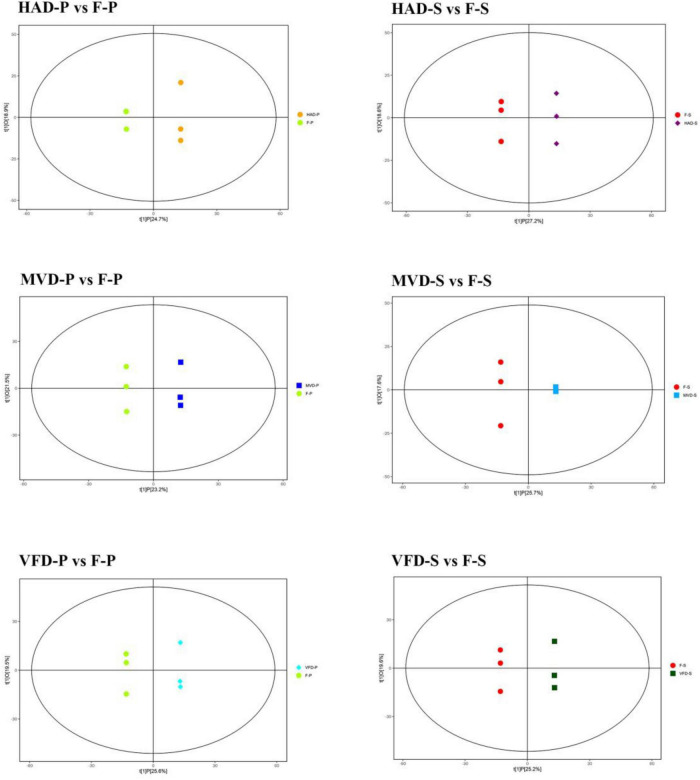
Score scatterplots of the fitted OPLS-DA models to the metabolite data.

To better understand the effect of each drying method upon the metabolism of *S. rugosoannulata*, the orthogonal partial least squares discriminant analysis (OPLS-DA) model was used to compare the metabolic characteristics of different samples. The OPLS-DA scatterplot scores of HAD-S vs. F-S, VFD-S vs. F-S, MVD-S vs. F-S, HAD-P vs. F-P, VFD-P vs. F-P, MVD-P vs. F-P comparison groups are shown in Figure 3. These results show that all the samples were within the 95% confidence interval (Hotelling’s T-squared ellipse), suggesting that HAD, VFD, and MVD are all significantly different from fresh *S. rugosoannulata*.

**FIGURE 4:**
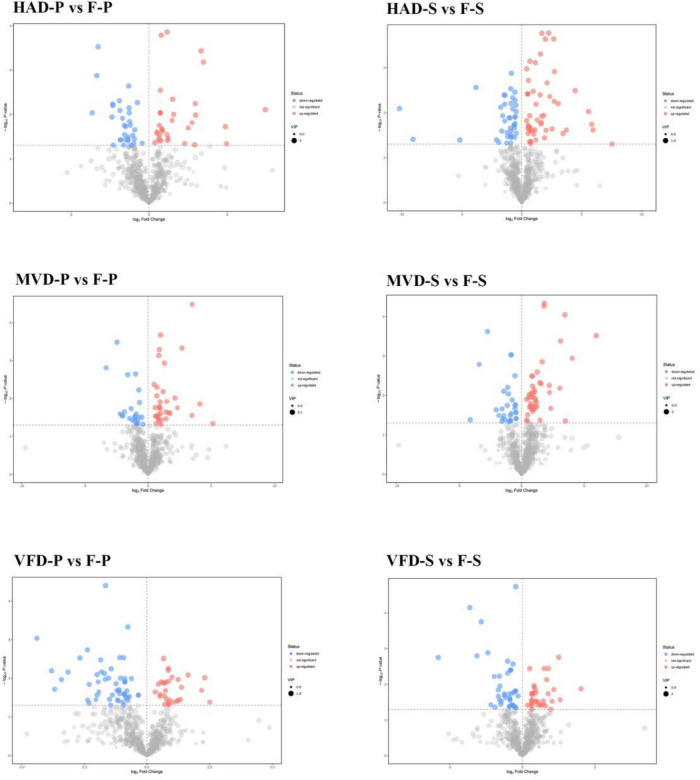
Volcano plots of differential metabolites in different comparison samples.

The analysis of Volcano plots was further applied to visualize the differences in the metabolites. In these plots, each point represents a metabolite and the scattered dots represent the VIP (Variable Importance in the Projection) values of the fitted OPLS-DA model. Scattered colors represent the final screening results, with significantly up-regulated (UR) metabolites in red, significantly down-regulated (DR) metabolites in blue, and non-significantly different metabolites in gray. Significant differential metabolites were designated according to the criterion of VIP ≥ 1 and *P* < 0.05. In the pairwise comparisons (Figure 4), 94 metabolites in HAD-S vs. F-S (53 UP and 41 DR), 78 metabolites in VFD-S vs. F-S (35 UP and 43 DR), 75 metabolites in MVD-S vs. F-S (44 UP and 31 DR), 66 metabolites in HAD-P vs. F-P (35 UP and 31 DR), 84 metabolites in VFD-P vs. F-P (31 UP and 53 DR), 59 metabolites in MVD-P vs. F-P (35 UP and 24 DR) were designated as significantly differentiated. Whether in the stipe or pileus, the quantity of significant differential metabolites in the MVD vs. F group was lower than that in the HAD vs. F group or VFD vs. F group; hence, the influence on metabolites from microwave vacuum drying is lower than that from the hot air drying and vacuum freeze drying methods.

**FIGURE 5:**
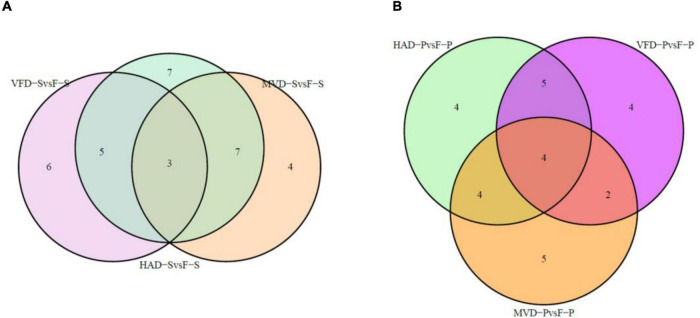
Venn diagrams of overlapping and unique non-volatile taste metabolites screened for differential expression. In panels **(A,B)** are the comparisons in stipe and pileus, respectively.

In order to further understand the impact of drying on flavor, we classified and compared the non-volatile flavor differential metabolites, including amino acid and derivatives, nucleotide and its derivatives, and organic acids and carbohydrates, produced under the different drying processes of *S. rugosoannulata*. Figure 5 illustrates that HAD-S vs. F-S and HAD-P vs. F-P respectively had 22 and 17 differential non-volatile taste metabolites, VFD-S vs. F-S and VFD-P vs. F-P respectively had 14 and 15 non-volatile taste differential metabolites, as did MVD-S vs. F-S and MVD-P vs. F-P. This indicated HAD induced more non-volatile flavor changes than did VFD and MVD. Notably, there was some overlap occurring among non-volatile flavor differential metabolites in those comparison groups. As seen in Table 1, after comparing the fresh and dried edible fungi, the main changes in their non-volatile flavor differential metabolites are amino acids and their derivatives, some of which are up-regulated while others are down-regulated. Results from earlier studies have suggested that the reason for irregular changes in amino acid content with drying time is that the degradation and synthesis of amino acids both occur throughout the drying process (31). In the HAD-S vs. F-S group and HAD-P vs. F-P group, evidently over half of the differentially metabolized amino acids and their derivatives were up-regulated. Yet in the VFD-S vs. F-S group and VFD-P vs. F-P group, less than one third of the differentially metabolized amino acids and their derivatives were up-regulated. For example, L-isoleucine, L-norleucine, and L-proline were up-regulated in both HAD-S vs. F-S and HAD-P vs. F-P groups, but down-regulated in the VFD-S vs. F-S and VFD-P vs. F-P groups. This suggests high temperatures promote protein degradation, consistent with the findings of Ai et al. (32). Although there were only five and four differentially metabolized amino acids and their derivatives in the MVD-S vs. F-S group and MVD-P vs. F-P group, the numbers of them up-regulated and down-regulated was relatively uniform. The same pattern was found for differential metabolites of nucleotide and its derivatives, organic acids and carbohydrates, most of them being up-regulated in HAD-S vs. F-S, HAD-P vs. F-P, MVD-S vs. F-S, and MVD-P vs. F-P groups yet often down-regulated in the VFD-S vs. F-S and VFD-P vs. F-P groups. For instance, adenine, cordycepin, 5-aminolevulinate, and D-xylulose were up-regulated in HAD-S vs. F-S, HAD-P vs. F-P, MVD-S vs. F-S, and MVD-P vs. F-P groups, but in VFD-S vs. F-S and VFD-P vs. F-P groups they were either down-regulated or showed no significant difference.

**TABLE 1:** Statistics for the differentially accumulated amino acid and derivatives, nucleotides and derivatives, carbohydrates, and organic acids during the different drying methods.

Class	Metabolites	HAD-S vs F-S				VFD-S vs F-S				MVD-S vs F-S				HAD-Pvs F-P				VFD-P vs F-P				MVD-P vs F-P			
		VIP	*p*	FC	Type	VIP	*p*	FC	Type	VIP	*p*	FC	Type	VIP	*p*	FC	Type	VIP	*p*	FC	Type	VIP	*p*	FC	Type
Amino acid and derivatives	2-Aminoisobutyric acid																	1.87	0.00	1.59	Up	2.01	0.00	1.81	Up
	4-Aminobutyric acid													1.04	0.10	2.21	Up					1.29	0.02	4.98	Up
	5-Aminovaleric acid					1.83	0.00	0.49	Down									1.76	0.02	0.47	Down				
	5-Oxoproline	1.77	0.01	0.73	Down																				
	Allysine(6-Oxo DL-Norleucine)																	1.78	0.01	1.37	Up				
	CYS-GLY;Cysteinyl glycine	1.90	0.04	0.03	Down					1.88	0.04	0.06	Down												
	DL-Norvaline	1.70	0.02	1.32	Up					1.63	0.04	1.37	Up												
	Glutathione oxidized													1.90	0.00	0.16	Down	1.32	0.01	0.26	Down				
	Guanidineacetic acid																	1.68	0.02	0.47	Down				
	Isoleucine	1.82	0.00	1.41	Up	1.88	0.00	0.57	Down					1.82	0.01	1.50	Up	1.83	0.01	0.54	Down				
	L-Aspartic acid	1.75	0.01	0.67	Down					1.70	0.03	0.73	Down	1.91	0.01	0.28	Down	1.81	0.01	1.81	Up	2.04	0.03	0.25	Down
	L-Glutamic acid					1.79	0.02	0.72	Down																
	L-Isoleucine; L-Leucine	1.82	0.00	1.41	Up	1.88	0.00	0.57	Down					1.82	0.01	1.50	Up	1.83	0.01	0.54	Down				
	L-Lysine; L-Glutamine	1.64	0.03	0.73	Down																				
	L-Norleucine	1.82	0.00	1.41	Up	1.88	0.00	0.57	Down					1.82	0.01	1.50	Up	1.83	0.01	0.54	Down				
	L-Pipecolic acid	1.72	0.02	0.65	Down					1.68	0.04	0.74	Down												
	L-Serine					1.78	0.04	0.67	Down									1.71	0.03	0.79	Down				
	L-Valine									1.65	0.04	1.31	Up												
	N,N-Dimethylglycine																	1.75	0.01	1.49	Up	1.89	0.01	1.62	Up
	Proline; L-Proline	1.65	0.03	1.60	Up	1.72	0.04	0.63	Down					1.72	0.02	1.59	Up	1.78	0.03	0.54	Down				
	S-Adenosylmethionine	1.92	0.01	0.00	Down																				
Nucleotide and its derivates	5′-Deoxyadenosine									1.76	0.00	8.62	Up												
	5′-S-Methyl-5′-thioadenosine													1.88	0.01	62.00	Up								
	Adenine	1.79	0.01	3.15	Up					1.70	0.02	2.33	Up	2.00	0.00	6.34	Up	1.67	0.03	0.59	Down	1.92	0.02	3.12	Up
	Adenosine					1.57	0.03	4.13	Up																
	Adenosine 5′-monophosphate													1.54	0.04	0.47	Down								
	beta-Nicotinamide mononucleotide	1.86	0.04	0.23	Down									1.82	0.03	0.42	Down					1.86	0.04	0.49	Down
	Cordycepin	1.91	0.01	8.18	Up	1.90	0.00	0.19	Down	1.95	0.00	3.46	Up	1.86	0.00	2.32	Up	1.57	0.01	0.20	Down	1.72	0.04	1.80	Up
	Cytidine	1.91	0.00	3.92	Up									1.73	0.03	1.91	Up					1.96	0.00	2.46	Up
	Cytidine 5′-monophosphate (Cytidylic acid)	1.79	0.02	61.35	Up					1.85	0.00	62.75	Up									1.75	0.01	17.11	Up
	Cytosine	1.70	0.01	6.53	Up									1.52	0.02	4.03	Up								
	Guanosine					1.75	0.04	0.27	Down																
	Guanosine 3′,5′-cyclic monophosphate	1.80	0.02	57.07	Up																				
	Pseudouridine																					1.70	0.00	0.10	Down
	Uridine 5′-diphospho-D-glucose;UDP-D-galactose; Uridine diphosphateg alactose	1.68	0.01	0.37	Down					1.71	0.02	0.69	Down	1.97	0.02	0.42	Down	1.70	0.03	1.45	Up	1.97	0.00	0.33	Down
	Uridine 5′-monophosphate									1.59	0.00	16.52	Up									1.76	0.00	11.26	Up
	Xanthosine													1.96	0.02	15.09	Up					1.92	0.01	4.37	Up
Organic acids	2-Hydroxybutanoic acid	1.78	0.01	1.58	Up					1.89	0.01	1.84	Up	1.67	0.04	1.43	Up								
	5-Aminolevulinate	1.87	0.00	1.61	Up	1.68	0.02	0.62	Down	1.83	0.01	1.49	Up	1.77	0.03	1.46	Up					1.94	0.00	1.39	Up
	Phosphoric acid	1.73	0.02	1.44	Up	1.91	0.00	1.41	Up	1.91	0.00	1.83	Up												
Carbohydrates	D-Xylulose																	1.91	0.03	0.54	Down				
	Sucrose	1.42	0.10	3.50	Up																				

These results indicated that the non-volatile flavor compounds of *S. rugosoannulata* underwent conversion during the different drying methods, especially in terms of differentially metabolized amino acids and nucleotides. Therefore, the non-volatile flavor of *S. rugosoannulata* after drying was probably related to the metabolism of amino acids and nucleotides. Sun et al. (7) argued that the umami taste of edible mushrooms is closely related to amino acid metabolism, nucleotide metabolism, and the Maillard reaction. Many umami amino acids and 5’-nucleotides in mushroom were investigated in numerous studies (25). This article not only focuses on these common non-volatile flavor substances, but also inferred the effects of related metabolites and derivatives, which is beneficial for comprehensively understanding the mechanism by a given drying method alters the metabolism of flavor-determining substances.

### Enrichment Analysis and KEGG Pathway Analysis of the Differential Metabolites

**FIGURE 6:**
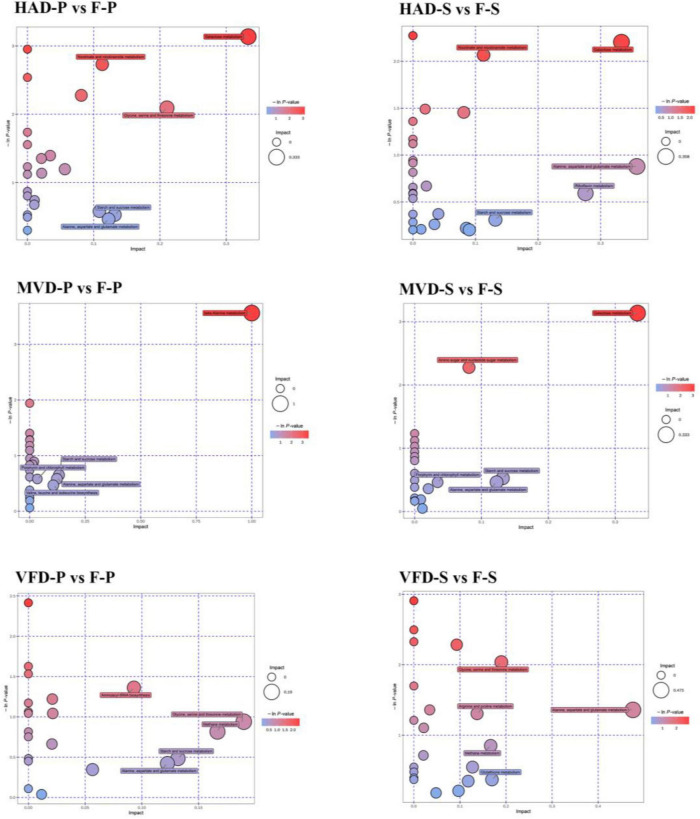
Metabolic enrichment pathway analysis.

Other researchers explored the main metabolic pathways of volatile flavor compounds in *shiitake* mushrooms through KEGG enrichment analysis: these being histidine metabolism, glutathione metabolism, and unsaturated fatty acid biosynthesis (33). However, only a few studies have investigated non-volatile flavors in mushrooms by conducting a widely targeted metabolomics analysis. In this study, the differential metabolites in fresh and dried samples were mapped to KEGG, PubChem, and HMDB online databases, and their enrichment results are shown in Figure 6. In it, metabolic pathway analyses are presented in bubble charts, in which each bubble denotes a metabolic pathway: the larger the bubble size, the greater the impact factor (based on a topological analysis); the darker the color, the smaller the *p*-value (enrichment analysis), and the more significant the degree of enrichment for that pathway.

The pathways of differential metabolites in the groups HAD-S and F-S were mainly concentrated in galactose metabolism; nicotinate and nicotinamide metabolism; alanine, aspartate, and glutamate metabolism; riboflavin metabolism; starch and sucrose metabolism. Those of the differential metabolites in the groups VFD-S and F-S were consisted chiefly of glycine, serine, and threonine metabolism; arginine and proline metabolism; alanine, aspartate, and glutamate metabolism; methane metabolism; glutathione metabolism. The pathways of differential metabolites in the groups MVD-S and F-S primarily comprised galactose metabolism; amino sugar and nucleotide sugar metabolism; starch and sucrose metabolism; porphyrin and chlorophyll metabolism; alanine, aspartate, and glutamate metabolism. Evidently, different drying methods can lead to disparate enrichment levels, whose difference also presented in the pileus. In this respect, pathway analysis revealed the enrichment in groups HAD-P and F-P being concentrated in galactose metabolism; nicotinate and nicotinamide metabolism; glycine, serine, and threonine metabolism; starch and sucrose metabolism; alanine, aspartate, and glutamate metabolism. By contrast, the metabolic pathways of differential metabolites in groups VFD-P and F-P mainly included aminoacyl-tRNA biosynthesis; glycine, serine, and threonine metabolism; methane metabolism; starch and sucrose metabolism; alanine, aspartate, and glutamate metabolism. The pathways of differential metabolites in groups MVD-P and F-P were principally involved in beta-alanine metabolism; starch and sucrose metabolism; porphyrin and chlorophyll metabolism; alanine, aspartate, and glutamate metabolism; valine, leucine, and isoleucine biosynthesis. Further, some metabolic pathways overlapped in these comparison groups, but their divergent enrichment levels provided compelling evidence that different modes of drying can produce differentially exclusive metabolites. Those changes in metabolic levels could be used to understand the impact of drying methods on *S. rugosoannulata.*

**FIGURE 7:**
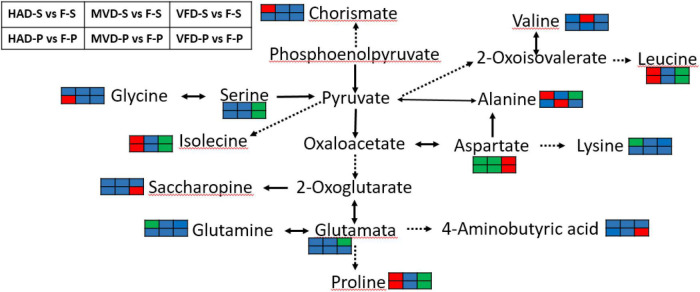
Biosynthesis of amino acids. (The red color rectangle indicates that the metabolite content is significantly up-regulated; the green rectangle indicates that the metabolite content is significantly down-regulated; the blue rectangle indicates no significant difference in that metabolite content).

Although the drying method differs, we can see that amino acid metabolism was the main metabolic pathway affected in all treatments. To gain insight into the effects of drying methods on amino acid metabolism in *S. rugosoannulata*, see Figure 7 based on the results from the KEGG annotation and enrichment analysis. Alanine, aspartate, and glutamate metabolism is clearly the common major metabolic pathway impacted by drying the stipe *via* hot air, freezing, or microwaves. Almost all amino acids take part in transamination reactions with pyruvate, oxaloacetate, or α-ketoglutarate to product alanine, aspartate, or glutamate, respectively (34). In addition, aspartate, glutamate, and alanine exert a great influence on both umami and sweetness of mushroom. Figure 7 verified that drying, especially by hot air, substantially impacts the flavor amino acids of *S. rugosoannulata* at the metabolic level. Under the action of amino acid metabolism, proline, leucine, and isolecine in the groups HAD-S vs. F-S and HAD-P vs. F-P were all up-regulated.

4-Guanidinobutyric acid has the physiological effects of improving the body’s sleep quality (35) and reducing blood pressure (36), had also been transformed in alanine, aspartate and glutamate metabolism. Notably, we found GABA up-regulated in groups VFD-P vs. F-P. It is known that GABA is a metabolic response to environmental stresses, for instance heat, cold and drought (37). Freeze-drying conditions could have up-regulated GABA in the pileus of *S. rugosoannulata*, probably due to glutamate being catalyzed by glutamate decarboxylase (EC:4.1.1.15) into GABA.

In the groups VFD-S vs. F-S and VFD-P vs. F-P, the common major metabolic pathway was that of glycine, serine, and threonine metabolism. Compared with hot air drying and microwave drying, the freeze drying mode has a greater impact on the metabolism of serine, and aspartate, which are the sweet and umami components in the un-volatile flavor profile of mushrooms. Serine was down-regulated in groups VFD-S vs. F-S and VFD-P vs. F-P, perhaps because its synthesis rate is slowed in a low-temperature drying condition. Li et al. (14) found that temperature could alter the rate of protein degradation into amino acids in *Pleurotus eryngii*. Therefore, it may be that the enzymatic reaction related to serine production is impaired and limited by freeze-drying, while the high temperature of hot air drying promotes protein degradation. Still, we did find several down-regulated metabolites after HAD, such as aspartate and lysine. The Maillard reaction is considered the paramount non-enzymatic reaction in the late stage of drying (38), one which could use amino acids to react with sugars. Moreover, the loss of moisture after drying can promote the Strecker degradation that would convert an amino acid into an aldehyde containing the side chain (39). MVD caused changes in starch and sucrose metabolism of *S. rugosoannulata*, which suggests microwave drying can elicit changes to sugar flavor substances. Nevertheless, because of the complicated reactions caused under the three drying methods, a comprehensive evaluation of the non-volatile flavor profile in dried *S. rugosoannulata*, such as its amino acids, nucleotides, organic acids, and soluble sugars, must be further investigated.

## Conclusion

In this study, a widely targeted metabolomics technology was applied to study the formation mechanism of non-volatile taste components obtained by different drying methods of the mushroom *S. rugosoannulata.* Overall, the results showed that the diversity of metabolites in the samples changed little in response to the three drying methods tested, but their relative content and metabolic pathways were significantly different. Comparing the three modes of drying, HAD is capable of improving both umami and sweetness by increasing the content of related amino acids and nucleotides, but the highest organic acid content is attained using MVD. We found the metabolite differences caused by microwave drying are less pronounced than those of HAD or VFD. Conversion of non-volatile taste compounds mainly occurs *via* the metabolism of amino acids and nucleotides. KEGG pathway analysis revealed that alanine, aspartate, and glutamate metabolism, these having a greater impact on umami, sweetness, and GABA, was the common major metabolic pathway affected after drying the stipe by HAD, VFD, and MVD. This study also shows that the metabolite differences caused by different drying methods are modulated by temperature, especially in that higher temperatures may cause protein degradation, the Strecker reaction, and the Maillard reaction. These findings indicate that in the actual production process, HAD, which is economical, convenient and better at improving the non-volatile taste components, could be given priority as a drying method. This study also advances our understanding of the transformation of nutrients and flavor substances in *S. rugosoannulata* caused by different drying methods. Nonetheless, further research is needed to elucidate the mechanisms underpinning these metabolism and molecular changes. Meanwhile, in order to fully explore the influence of drying methods on the flavor of *S. rugosoannulata*, the volatile flavor substances and other substances that may affect the flavor should also be analyzed qualitatively or quantitatively.

## Data Availability Statement

The data presented in the study are deposited in the MetaboLights repository, accession number MTBLS4460 (www.ebi.ac.uk/metabolights/MTBLS4460).

## Author Contributions

YL: conceptualization, methodology, formal analysis, writing—original draft, and writing—review and editing. FM: data curation and writing—original draft. PT: supervision and writing—review and editing. DH: software and project administration. QL: investigation and validation. ML: supervision, writing—review and editing, funding acquisition, and data curation. All authors contributed to the article and approved the submitted version.

## Conflict of Interest

YL and ML were employed by Guizhou Characteristic Food Technology Co., Ltd. The remaining authors declare that the research was conducted in the absence of any commercial or financial relationships that could be construed as a potential conflict of interest.

## Publisher’s Note

All claims expressed in this article are solely those of the authors and do not necessarily represent those of their affiliated organizations, or those of the publisher, the editors and the reviewers. Any product that may be evaluated in this article, or claim that may be made by its manufacturer, is not guaranteed or endorsed by the publisher.
